# Acute Effects of Cannabis and THC Oils on Cardiovascular Hemodynamics and Muscle Electrical Activity in Healthy Individuals: A Controlled Clinical Crossover Trial Protocol

**DOI:** 10.3390/jcm14217531

**Published:** 2025-10-24

**Authors:** Marina Lyra Lima Cabral Fagundes, Edna Karla Ferreira Laurentino, Bruno Lobão Soares, Matías Otto-Yañez, Emerson Arcoverde Nunes, Matheus de Freitas Fernandes Pedrosa, Jessica Danielle Medeiros da Fonseca, Vanessa Regiane Resqueti, Guilherme Augusto de Freitas Fregonezi

**Affiliations:** 1 Laboratório de Inovação Tecnológica em Reabilitação, Departamento de Fisioterapia, Universidade Federal do Rio Grande do Norte, Natal 59078-970, Brazilvanessa.resqueti@ufrn.br (V.R.R.); 2 PneumoCardioVascular LAB/HUOL, Departamento de Fisioterapia, Hospital Universitário Onofre Lopes, Universidade Federal do Rio Grande do Norte, Natal 59078-970, Brazil; 3 Departamento de Biofísica e Farmacologia, Universidade Federal do Rio Grande do Norte, Natal 59078-970, Brazil; 4 Grupo de Investigación en Salud Funcionalidad y Actividad Física (GISFAF), Kinesiologia, Facultad de Ciências de la Salud, Universidad Autônoma de Chile, Santiago 8910060, Chile; 5 Hospital Universitário Onofre Lopes, Universidade Federal do Rio Grande do Norte, Natal 59078-970, Brazil; 6 Laboratório Farmacêutico de Tecnologia e Biotecnologia, Departamento de Farmácia, Universidade Federal do Rio Grande do Norte, Natal 59078-970, Brazil

**Keywords:** *Cannabis sativa*, endocannabinoids, hemodynamic monitoring, randomized controlled trial, electromyography

## Abstract

**Background/Objectives: ***Cannabis sativa* (CS) exerts its effects through the endocannabinoid system. Although studies have shown limited evidence regarding the plant’s therapeutic efficacy, little is known about the standardization of doses and their corresponding effects. This study aims to analyze changes in muscle electrical activity and cardiovascular hemodynamics before, during, and after administering doses of full-spectrum cannabis and tetrahydrocannabinol (THC) oils. **Methods:** Participants will be assigned to a single group that will undergo five different interventions: CBD + THC at 12.50 mg, CBD + THC at 18.75 mg, THC intervention at 12.50 mg, THC at 18.75 mg, and placebo intervention (PI). The study will enroll healthy, self-reported men and women aged 18 to 50, with no mental health disorders and no exposure to CS in the past six months. Interventions will occur on five randomized days, following three phases: (1) pre-intervention (PRE-IN); (2) intervention (IN)—1 h after oral oil ingestion; and (3) post-intervention (POST-IN)—2 h 30 min after ingestion. At each stage, 2 min of quiet breathing (QB); 2 min with an inspiratory resistance valve (30% of maximal inspiratory pressure—PImax) and expiratory valve (10% of maximal expiratory pressure—PEmax) (VAL); and 4 min of recovery without the valve (REC) were evaluated. Register: RBR-3jsvtbr. **Conclusions:** This study will enhance the understanding of the dose–response effects of full-spectrum cannabis and THC oils and may serve as a model for future research in this field.

## 1. Introduction

The *Cannabis sativa* (CS) plant, commonly known as marijuana, is an illicit drug widely used throughout the world, primarily for recreational purposes. Its use dates back to the third millennium B.C. in India, where it was used primarily for medicinal purposes, including the treatment of malaria, constipation, pain, and dysmenorrhea. Over the centuries, its use spread throughout Central Asia and was included in various pharmacopeias worldwide, covering regions such as Asia, the Middle East, South Africa, and South America [1,2].

A major milestone in the history of cannabis occurred in 1964 with the isolation of its main active ingredient, delta-9-tetrahydrocannabinol (Δ9-THC). Prior to this, research on the plant was limited due to high variability in dosage and, consequently, its therapeutic effects. During this period, the plant was classified as dioecious, with the female flower identified as the main source of cannabinoids, from which various substrates are extracted to produce medicines [3].

The number of phytocannabinoids in CS is about 100, with the most well-known being Δ9-THC and cannabidiol (CBD) [4]. The effects of these compounds on the human body are attributed to the complex endocannabinoid system, which includes not only endocannabinoids synthesized internally but also cannabinoid receptors type 1 (CB1) and type 2 (CB2) present in various cells throughout the body. It also affects the enzymes responsible for the production and degradation of endocannabinoids [5,6]. THC acts as a partial agonist at CB1 and CB2 receptors within the endocannabinoid system, exerting its psychoactive and pain-modulating effects primarily through CB1 activation. In contrast, CBD has low affinity for the active sites of these receptors [7,8]. Therefore, CB1 receptors appear to be the most relevant for changes in muscle electrical activity and cardiovascular hemodynamics, as they are primarily located in the central nervous system and modulate both neural control of muscle contraction and autonomic cardiovascular responses.

Extracted from CS, tetrahydrocannabinol (THC) oil has been shown to interact with receptors present in the brain, significantly impacting the development of cannabinoid-based therapeutic drugs and their potential to alleviate symptoms of spasticity [9]. Cannabidiol (CBD) oil, on the other hand, is a non-psychoactive compound used in medicine for its antiepileptic, anti-inflammatory, antiemetic, antipsychotic, anxiolytic, and muscle relaxant effects [10].

Several studies suggest that CBD may offer therapeutic benefits in a wide range of conditions, mental psychological disorders, diabetes, gastrointestinal problems, cancer, oxidative stress, inflammation, and cardiovascular disease [11,12,13]. Also has shown that an acute dose of CBD can cause vasodilation and reduce blood pressure in humans, possibly through the activation of CB1 receptors [14]. In addition, CBD has been associated with improved myocardial dysfunction in models of diabetes [15]. However, there is still a significant lack of human studies investigating the effects of CBD on respiratory muscle and cardiovascular hemodynamics.

Research using lung tissue found that THC induced a concentration-dependent inhibition of cholinergic contraction in human airway smooth muscle cells through the activation of prejunctional CB1 receptors [16]. Based on this finding, Morris and coworkers [17] found a significant association between inhaled cannabis use and improvements in forced expiratory volume in the first second (FEV_1_) and forced vital capacity (FVC) in individuals with COPD.

Abdallah and collaborators [18] conducted a study to investigate whether a single dose of vaporized inhaled cannabis could relieve exertional breathlessness and improve exercise endurance, focusing on improving static and dynamic airway function in adults with advanced COPD. However, the results did not show any significant effects on lung function measured by spirometry, nor on exertional breathlessness during effort or exercise endurance capacity.

Overall, information on the pharmacokinetics and pharmacodynamics of cannabis-derived medications remains limited. However, a recent study using Spectrum Yellow oil 20 mg/mL of cannabidiol (CBD)/0.9 mg/mL Δ9-tetrahydrocannabinol (THC) found that a prudent approach to assessing tolerability includes initial doses of 240 mg of CBD and 10.8 mg of THC [19]. These findings highlight the importance of further research to better understand the effects of these compounds, thereby contributing to a safer and more effective approach to the use of cannabis-derived medications.

In addition, there is a lack of standardization regarding dosage and routes of administration. The drug exhibits high interindividual pharmacokinetic variability when administered orally, which may be influenced by demographic factors such as sex and weight [20]. These factors hinder the standardization of cannabis use and the identification of tolerability parameters, underscoring the need for studies comparing different doses and their effects in humans.

To achieve this goal, our study proposes a comprehensive approach designed to describe the potential changes and investigate the dose–response relationship to the acute effects of cannabis and THC oils in full-spectrum extraction. This study will be conducted in healthy individuals subjected to unidirectional respiratory overload and will assess cardiovascular hemodynamics and respiratory muscle electrical activity, allowing for a detailed examination of the effects of these compounds on different body systems and thus providing a more comprehensive understanding of their physiological effects. These data are crucial for future research and the development of treatments related to respiratory, cardiovascular, and muscular rehabilitation associated with medical cannabis. They also offer valuable insights into how these compounds can be used safely and effectively in clinical and therapeutic settings.

## 2. Materials and Methods

This is a randomized controlled crossover clinical trial with a 1:1:1 allocation ratio, double-blinded, which will be described according to the Consolidated Standards of Reporting Trials (CONSORT) criteria [21] where the checklist will be available in the online Supplementary Materials, and the protocol will follow the description suggested by the Standard Protocol Items Recommendations for Interventional Trials (SPIRIT) [22]. This study will be conducted at the Onofre Lopes University Hospital, of the Federal University of Rio Grande do Norte. The study was authorized by Brazilian Health Regulatory Agency (ANVISA) and was registered in the Brazilian Clinical Trials Registry—ReBEC, a public registry, under the trial registration number RBR-3jsvtbr, with the registration date of 30 April 2024.

### 2.1. Sample Size and Allocation

The sample size will be determined after a pilot study with five subjects, considering the cardiac output (CO) values as the main variable in intra- and inter-group comparisons through mixed model analysis of the Generalized Estimating Equations (GEE) test. An alpha error of 0.05 with a two-sided distribution and a test power of 80% will be established. Recruitment will be done through convenience sampling, using direct invitations and advertising on social media, posters, flyers on Instagram and WhatsApp, targeting the general population.

Participants will be randomly assigned to a single group, which will undergo five different types of interventions: (1) CBD + THC intervention at 12.50 mg (CBD + THC/12.50 mg), (2) CBD + THC at 18.65 mg (CBD + THC/18.65 mg), (3) THC intervention at 12.50 mg (THC/12.50 mg), (4) THC at 18.65 mg (THC/18.65 mg), and (5) placebo intervention (IP). The order of interventions for each individual will be randomized using the https://www.sealedenvelope.com website for analysis (accessed on 4 September 2025).

To conduct the five research interventions, we will use three different oils. The first is an THC oil with a concentration of 2.50 mg/mL “Naternal Full Spectrum Delta 9 THC”, from (Naternal^®^, Morrisville, NC, USA). The second is a Cannabis oil containing 50 mg/mL CBD and 1.83 mg/mL THC “Lazarus Naturals High Potency Full Spectrum” from (Lazarus Naturals^®^, Seattle, DC, USA). The third, used in the placebo intervention (IP), is an MCT (Medium Chain Triglycerides) oil used as a base for diluting the full-spectrum Cannabis and THC oils, consisting of 70% fractionated coconut oil and 30% corn oil.

During the THC evaluations, the mL dosages will be determined based on the concentration of the product. Similarly, in the evaluations of the CBD + THC intervention, the THC and cannabis oils will be combined to achieve an equal ratio of CBD and THC for each specific dose. In the placebo intervention, all participants will receive a dose of 5.00 mL of MCT. The dosages and corresponding values are detailed in Table 1.

### 2.2. Eligibility Criteria

The study will include self-reported healthy Brazilian adults aged 18 to 50 years, with a body mass index (BMI) between 18.5 and 29.9 kg/m^2^, without respiratory dysfunction (i.e., forced vital capacity [FVC] > 80% and a ratio of forced expiratory volume in one second [FEV_1_] to FVC > 0.7 or >85% of predicted) [23], no uncontrolled or untreated mental disorders, no diagnosed cardiovascular disease, no history of arrhythmias or hypertension, no regular medication use, and no cannabis exposure in the past six months, and no history of substance abuse.

Participants will be excluded from the study if they withdraw, are unable to understand or complete any part of the study, or exhibit clinical instability during the procedures (i.e., heart rate > 85% of maximum heart rate, hypertensive peak with systolic blood pressure > 180 and/or diastolic blood pressure > 110 mmHg, hypertensive crisis with blood pressure > 190/90 mmHg or 175/115 mmHg). Pregnant women are also excluded.

### 2.3. Sample Allocation Procedure and Allocation Concealment

All participants will be assigned to a single group, with the order of intervention randomly assigned through a one-group, five-arm randomization process known only to one researcher (Evaluator 1). This researcher will be responsible for delivering the order in which the three bottles, identified by stickers on the bottom, will be applied by the second researcher (Evaluator 2), who will be responsible for applying the intervention protocols and collecting data. These bottles, containing the oil corresponding to the allocation group, will be numbered 1 to 3. The contents of these bottles, identical in color and viscosity, will be known only to the researcher responsible for randomization (Evaluator 1), who will remain locked in a room throughout the data collection. After all stages of the intervention: 1-Pre-Intervention (PRE-IN); 2-Intervention (IN); 3-Post-Intervention (POST-IN)—the data collection will be assessed by the third researcher (Evaluator 3). Ensure allocation concealment and blinding of both participants and researchers.

### 2.4. Study Design

After agreeing to participate in the study and signing the Informed Consent Form (ICF), data collection will begin over five days, during which the administration of CBD + THC, THC, both at two different dosages, or MCT oil, all will be evaluated in three stages: 1-Pre-Intervention (PRE-IN); 2-Intervention (IN); 3-Post-Intervention (POST-IN).

Because hemodynamic variables can be affected by medications, supplements, caffeine, smoking, and temperature [24,25], participants are asked to refrain from taking supplements for 72 h prior to the IN and to avoid exercise, caffeine, alcohol, and smoking for 24 h prior to the IN. In addition, the IN and POST-IN stages will always be performed in the same time period (afternoon) to avoid circadian influences.

The first day will be divided into two sessions: the morning session will include randomization, baseline assessment, and PRE-IN assessment. First, participants will be instructed to sit down and complete a self-administered 14-item Hamilton Anxiety Scale (HAM-A). Anthropometric data will then be collected by measuring the participant’s weight on a scale, height using a stadiometer, and blood pressure using an automated digital cuff.

Surface electromyography (sEMG) electrodes are placed on the respiratory muscles and remain in place throughout the data collection. Then, maximum inspiratory pressure (PImax) and maximum voluntary ventilation (MVV) assessments will be performed to normalize the electromyography data collected at all assessment stages. Baseline data will then be collected to characterize the sample, including spirometry and digital manometry to measure respiratory muscle strength (PImax and PEmax) and lung function.

Next, the PhysioFlow^®^ (Manatec Inc., Paris, France). electrodes will be placed, and the NIRS device will be attached to the participant, who will then be taken to bed to begin the pre-intervention stage (PRE-IN). In a semi-recumbent position with the trunk elevated at 45°, hemodynamics will be evaluated by electrical impedance through PhysioFlow^®^, respiratory muscle activity will be measured by Surface Electromyography (sEMG), and tissue oxygenation will be assessed via near-infrared spectroscopy (NIRS). After this evaluation stage, which will be used for comparison with the IN and POST-IN stages of the other interventions.

**Figure 1:**
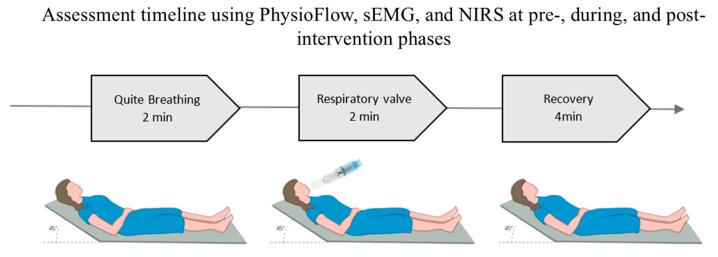
Data collection positioning during assessment moments.

For all stages of respiratory Intervention (PRE-IN, IN and POS-IN) the data will be recorded for 8 min: 2 min of quiet breathing (QB), 2 min of breathing with inspiratory resistance at 30% of PImax and expiratory resistance at 10% of PEmax (VAL) using the BigBreathe^®^ valve (IMT/PEP, GHINNOTEK, Busan, Republic of Korea), followed by 4 min of recovery with spontaneous breathing (REC), as illustrated in Figure 1. During the 8 min of data collection, systolic blood pressure (SBP) and oxygen saturation (SaO_2_) will also be assessed, in addition to the Borg scale for mouth fatigue and dyspnea.

**Figure 2:**
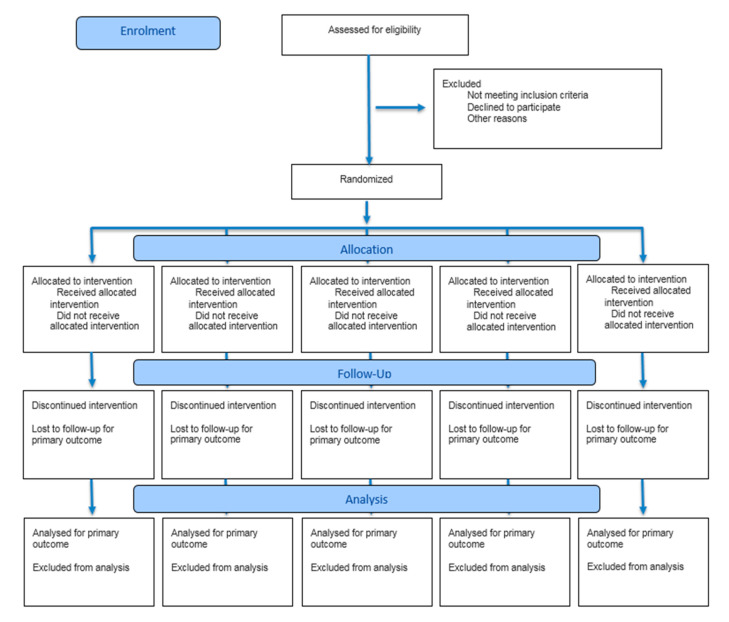
CONSORT 2025 Flow Diagram.

Except for the baseline stage, the other evaluation moments will take place exclusively in the afternoon. The study design can be better observed in Figure 2. Upon arriving at the research site, the participant will sit for the placement of electrodes and will then be taken to a bed, where they will be informed about the start of the pharmacological intervention. The respective oil, previously delivered by the responsible researcher following randomization, will be administered. Under the researcher’s guidance, the participant will be asked to wear a blindfold to cover their eyes and use a nasal clip to avoid any identification of the oil administered, open their mouth, and lift their tongue for the dose in drops. After the administration, the researcher will instruct the participant to remain seated and at rest. After 60 min, the second stage (IN) will take place and after 2 h and 30 min post-administration of the drug, the third evaluation stage (POST-IN) will begin.

The BigBreathe^®^ device will allow bidirectional threshold overload (IMT/PEP, GHINNOTEK, Busan, Republic of Korea) with an IMT range of 10–40 cmH_2_O and PEP of 5–20 cmH_2_O, with a resolution of 2 cmH_2_O. A variable load can be set on the IMT/PEP device, providing independent resistance to inspiration or expiration using two spring-loaded one-way valves. These valves only open when the pressure generated by the individual exceeds the spring tension set during inspiration and expiration, with simultaneous IMT and PEP effort in a single respiratory cycle [26]. A load of 30% of PImax and 10% of PEmax will be established for each participant, as previously described, as initial loads for respiratory muscle training, aiming only to activate the musculature while also considering the participant’s 45° trunk position.

### 2.5. Outcomes

#### 2.5.1. Primary Outcome

Hemodynamic evaluation will be performed using the PhysioFlow^®^ device (Manatec Inc., Paris, France). It is a non-invasive cardiac output monitor that provides hemodynamic parameters through transthoracic electrical bioimpedance signal analysis. PhysioFlow measures changes in impedance by applying a high-frequency alternating current (75 KHz) with low amplitude (3.8 mA) through the chest. Two sets of electrodes (PhysioFlow OS-50, Manatec Biomedical, Macheren, France)—one transmitting and the other receiving the electric current—will be applied above the supraclavicular fossa at the left base of the neck and along the spinal line at the thoracolumbar transition. An additional pair will be used for continuous electrocardiographic monitoring, recorded as recommended by the manufacturer after shaving and cleaning the skin with alcohol, followed by abrasion with Nuprep^®^ (Weaver and Company, Aurora, CO, USA) gel.

The sEMG is used to acquire and process respiratory muscle activity, a non-invasive technique performed with the BTS FreeEMG 1000 (Milan, Italy) device from BTS Bioengineering, which performs all amplification and filtering signal processing. The signal is acquired using 4 sensors connected to 4 disposable electrodes (4 cm long, 2.1 cm wide and 2 cm between poles) placed on each muscle to be analyzed after proper skin preparation according to the appropriate positioning in the literature for better signal acquisition. The skin preparation will be done by shaving, cleaning with 70% alcohol, rubbing the skin with gauze and applying the electrodes in the direction of the muscle fibers, according to the SENIAM recommendations (1999) [27]. The analyzed muscles are: sternocleidomastoid (SCM), scalene (ESC), parasternal (PARA) and rectus abdominis (RA), all on the right side, positioned as follows at the lower third of the distance between the mastoid process and the sternoclavicular joint [28]; 5 cm from the sternoclavicular joint and 2 cm above this point [29]; at the second intercostal space and 3 cm from the sternum at the umbilical line, 4 cm from the umbilicus [30]. The data are acquired and stored by the BTS FreeEMG 1000 (BTS Bioengineering), which handles all amplification and filtering of the signal acquired by the 4 sensors. The collected signals are stored using the SmartCapture software (BTS Bioengineering, Milan, Italy) and analyzed using the SmartAnalyzer program version 1.10.470.0.

The analysis of the myoelectric signal is performed using the Root Mean Square (RMS), which evaluates the amplitude of the signal in microvolts. For the normalization of the sEMG signals of the SCM and ESC muscles, the electromyographic values of the PImax maneuver will be analyzed, and for the PARA muscle, the MVV maneuvers, while the normalization of the RA muscle signal will be obtained from the Maximum Voluntary Isometric Contraction (MVIC) maneuver.

The recorded sEMG signals are pre-processed with a Butterworth bandpass filter from 20 to 400 Hz. To filter the cardiac signal, we will use a Butterworth high-pass filter of 20 Hz. The use of electromyographic signal processing techniques such as filtering, smoothing, and rectification will precede the calculation of the mean value, which will then be adjusted as %RMS according to the normalization for each subject.

#### 2.5.2. Secondary Outcomes

Anthropometric data (height, weight, BMI) and an initial assessment form including age, sex, marital status, occupation, smoking, alcohol use, medication use, sleep duration in hours per day, presence of chronic diseases, heart disease, lung disease, psychological or psychiatric diagnosis, and previous cannabis use, including oil, will be collected from all subjects.

The initial assessments will include a clinical evaluation, covering vital signs (temperature, pulse, blood pressure, respiration, and heart rate) as well as the Hamilton Anxiety Scale (HAM-A) (Hamilton M, 1959) [31] to assess the presence and severity of anxiety symptoms in participants. The HAM-A consists of 14 items, each defined by a set of symptoms that measure both psychological anxiety (restlessness and psychological distress) and somatic anxiety (physical complaints). Each item is scored on a scale from 0 (none) to 4 (severe), with a total score ranging from 0 to 56, with a score below 17 indicating mild anxiety, between 18 and 24 indicating moderate severity, and between 25 and 30 indicating moderate to severe anxiety [31].

The Borg scale and dyspnea are also used, along with other monitoring measures such as respiratory rate (RR), heart rate (HR), and peripheral oxygen saturation (SpO2), which are monitored and recorded every 2 min during the PRE-IN, IN, and POST-IN phases.

During spirometry, the following variables are evaluated: Forced vital capacity (FVC), forced expiratory volume in one second (FEV_1_), forced expiratory flow between 25% and 75% of the FVC curve (FEF_25–75%_), and FEV_1_/FVC ratio. The evaluation is considered complete when three acceptable curves have been performed with two reproducible results according to the American Thoracic Society (ATS) and European Respiratory Society (ERS) guidelines [32]. The equipment used is a KoKo DigiDoser^®^ digital spirometer (Longmont, CO, USA) connected to a computer with the device’s own software, and calibration is performed daily by injecting a volume of air with a 3-L calibration syringe. The best spirometric values are selected for analysis. The values obtained will be compared with the relative and absolute values for the Brazilian population [33].

Inspiratory and expiratory muscle strength will be assessed by maximum respiratory pressures (and PEmax, respectively). The measurement will be performed with the digital manometer MicroRPM^®^ (Micro Medical, Rochester, UK). Prior to each test, participants will be instructed in the procedures and the assessors will demonstrate how to perform the maneuver. Participants will perform a trial maneuver for learning purposes and the assessment will be considered complete when there is less than 10% variation between the two maximum values. The technical criteria for acceptability and reproducibility of the maximum airway pressure tests follow the ATS/ERS guidelines [34].

Tissue oxygenation is assessed by near-infrared spectroscopy (NIRS) using the NIRSPortamon^®^ device (Artinis Medical Systems, BV, Elst, The Netherlands). The technique is based on the application of light at a near-infrared wavelength, taking into account the absorption and scattering principles based on spatially resolved spectroscopy. The NIRSPortamon^®^ is a non-invasive, portable, wireless device with a receiver and three LEDs spaced 30, 35 and 40 mm apart that detect the absolute concentrations of oxyhemoglobin (O_2_Hb), deoxyhemoglobin (HHb), total hemoglobin (tHb) and oxygen index, blood volume and flow. The device uses wavelengths of 760 and 850 nm and the Tissue Saturation Index (TSI) in muscle tissue, providing information on local consumption. A Bluetooth connection allows online monitoring while the individual is performing activities and is not affected by the presence of equipment such as sEMG and sECG. The device is attached to the individual’s skin over the medial vastus muscle using adhesive tape after shaving and cleaning the area with 70% alcohol. Data analysis was performed using Oxysoft software version 4.0 (Portamon Artinis Medical Systems, BV, Elst, The Netherlands).

### 2.6. Monitoring and Adverse Effects

This study is a Phase 1 research project; therefore, no previous studies have reported serious adverse effects from the use of CBD or THC oil. Potential pharmacological effects will be monitored throughout the study, and in anticipation of reactions such as allergic reactions or exacerbated effects, the intervention will take place in a hospital setting with a multidisciplinary health care team. The investigators are committed to providing appropriate and prompt treatment for any adverse events that may occur during the study. In the case of serious adverse events or effects, a final decision will be made to discontinue the study. In case of psychiatric symptoms, the participant will be accompanied by a psychiatrist and will receive pharmacological treatment if necessary.

### 2.7. Statistical Analysis

Normality and distribution of data will be assessed using the Shapiro–Wilk test. For descriptive analysis, we will use mean and standard deviation for parametric data and median and interquartile range for nonparametric data. Intra- and inter-group comparisons will be made using mixed model analysis, specifically the Generalized Estimating Equations (GEE) test, with adjustment for multiple comparisons using the Sequential Bonferroni correction in IBM SPSS Statistics version 25 (International, based in Armonk, NY, USA). Sample characterization data will be analyzed using GraphPad Prism 8.0 (GraphPad Software Inc., San Diego, CA, USA) for graph construction. A significant level of *p* < 0.05 and a 95% confidence interval (CI) are used for all analyses.

## 3. Discussion

This will be a randomized, double-blind, controlled, crossover study to be conducted in a hospital setting, aiming to evaluate the acute effects of different doses of full-spectrum cannabis and THC oils on cardiovascular hemodynamics and respiratory muscles’ electrical activity in healthy subjects. The use of cannabis oil, especially in the form of full-spectrum extracts or THC, has attracted increasing interest in various health fields. Although most studies have focused on the effects of these compounds on the central nervous system [35,36] and specific neurological conditions [12,37,38], there is a gap in knowledge regarding their physiological effects on the cardiovascular and respiratory systems, as well as their tolerability and the recommended doses for acute effects.

Stanley [39] suggests, in a review, that administered CBD does not appear to have any effect on resting blood pressure or heart rate; however, it reduces the cardiovascular response to various types of stress. Thus, CBD treatment may play a protective role in reducing the effects of cardiac ischemia and reperfusion, or in mitigating cardiac dysfunction associated with diabetes. On the other hand, Jadoon et al. [40] observed that a single oral dose of CBD reduced resting blood pressure compared to the placebo group. These responses may be related to the pharmacokinetic variability of effects, as well as the different doses and routes of administration used.

Pickering et al. [41] evaluated 9 healthy individuals and individuals with COPD (Chronic Obstructive Pulmonary Disease) who were subjected to dyspnea induced by inhalation of different loads of carbon dioxide (CO_2_). The research participants underwent an evaluation with an intervention using an oromucosal cannabis spray containing 2.7 mg of THC and 2.5 mg of CBD, where the maximum dose was 4 sprays. Despite the small number of participants evaluated, the study demonstrated that cannabis may attenuate descriptors of dyspnea, potentially detecting an improvement in the unpleasantness of induced dyspnea when compared to the placebo group, especially in subjects with COPD. However, it is still necessary to better understand the physiological response of the drug in the cardiovascular and respiratory systems to effectively use it in the treatment of diseases and dysfunctions.

Changes in respiratory muscle activity can significantly affect lung capacity, leading to symptoms such as dyspnea, fatigue, and exercise intolerance. The results of this research could provide relevant scientific evidence on the effects of cannabis oils and THC at different doses on the respiratory muscular system, and this information may have important clinical implications, helping in the evaluation of risks and benefits associated with the use of these substances, as well as in the development of personalized therapeutic protocols for patients with specific respiratory conditions.

Seeking to better describe these pharmacological effects, Nitecka-Buchta et al. [42] evaluated the muscle relaxant aspects of cannabis through sEMG in the masseter muscle in a double-blind clinical trial. And observed that, when applied transdermally in the form of an ointment, cannabis reduced electrical activity and pain intensity, which was beneficial for the group with temporomandibular dysfunction. However, further studies are needed to assess the effects of oral cannabis use on the electrical activity of respiratory muscles and its influence on breathing.

Therefore, by investigating the effects of both oils at varying doses in the healthy population, it is possible to gain a more precise understanding of the direct impacts of these substances on cardiovascular hemodynamics and respiratory muscle electrical activity. Furthermore, it may provide important insights for other target populations, such as those with neurological, respiratory, or cardiac diseases. Additionally, this study aims to expand scientific knowledge and could serve as a foundation for future studies exploring the therapeutic potential of these substances in various health conditions, offering valuable information for clinical decision-making and contributing to the advancement of knowledge in this field.

## 4. Conclusions

This study aims to deepen the understanding of the dose–response relationship of both full-spectrum cannabis oil and THC oil, contributing valuable insights into their physiological and potentially therapeutic effects. The findings may not only clarify the differential impacts of these compounds but also offer a structured framework for future investigations in the area of cannabinoid-based interventions. By establishing methodological guidelines and highlighting key outcome measures, this research could serve as a foundational reference for clinical and experimental studies moving forward.

## Figures and Tables

**Table 1:** This table summarizes the amount and type of substance used in each intervention arm of the research.

Substance	Intervention Arms				
	CBD + THC 12.50 mg	CBD + THC 18.75 mg	THC 12.50 mg	THC 18.75 mg	IP
Oil 1 THC Full-Spectrum (2.50 mg/mL THC)	4.81 mL	7.22 mL	5.00 mL	7.50 mL	-
Oil 2 Cannabis Full-Spectrum (50 mg/mL CBD + 1.83 mg/mL THC)	0.25 mL	0.37 mL	-	-	-
Oil 3 MCT (70% coconut 30% corn)	-	-	-	-	5 mL
Total (mL)	4.79 mL	7.19 mL	5.00 mL	7.50 mL	5 mL

## Supplementary Materials

The following supporting information can be downloaded at: https://www.mdpi.com/article/10.3390/jcm14217531/s1, Table S1: CONSORT 2010 checklist of information to include when reporting a randomised trial. Reference [43] is cited in the Supplementary Materials.

## Abbreviations

The following abbreviations are used in this manuscript:

BMI	Body mass index
CBD	Cannabidiol
COPD	Chronic Obstructive Pulmonary Disease
CS	Cannabis Sativa
ESC	Scalene
FEF_25–75%_	Forced expiratory flow between 25% and 75% of the FVC curve
FEV_1_	Forced expiratory volume in the first second
FVC	Forced vital capacity
GEE	Generalized Estimating Equations
HAM-A	Hamilton anxiety scale
HHb	Deoxyhemoglobin
ICF	Informed consent form
IN	Intervention
MCT	Medium Chain Triglycerides
MVIC	Maximum Voluntary Isometric Contraction
MVV	Maximum Voluntary Ventilation
NIRS	Near-infrared spectroscopy
O_2_HB	Oxyhemoglobin
PARA	Parasternal
PEMáx	Maximum expiratory pressure
PI	Placebo Intervention
PIMax	Maximum inspiratory pressure
POST-IN	Post-intervention
PRE-IN	Pre-intervention
QB	Quiet breathing
RA	Rectus abdominis
REC	Recovery
RMS	Root Mean Square
SaO_2_	Oxygen saturation
SBP	Systolic Blood Pressure
SCM	Sternocleidomastoid
sEMG	Surface electromyography
THC	Tetrahydrocannabinol
tHb	Total Hemoglobin
TSI	Tissue Saturation Index
VAL	Valve
